# Identification and comparison of biological characteristics and pathogenicity of different mating types of *V. dahliae* isolated from potato and sunflower

**DOI:** 10.1038/s41598-022-17196-x

**Published:** 2022-07-27

**Authors:** NingNing Yan, Mandela Elorm Addrah, Yuanyuan Zhang, Ruifang Jia, Liru Kang, Jun Zhao, Jian Zhang

**Affiliations:** 1 College of Horticulture and Plant Protection, Inner Mongolia Agricultural University, Hohhot, 010018 China; 2 Institute of Grassland Research of Chinese Academy of Agricultural Sciences, Hohhot, 010010 China; 3 Inner Mongolia Academy of Agronomy and Animal Husbandry Sciences, Hohhot, China

**Keywords:** Fungal pathogenesis, Fungal biology

## Abstract

Potato is one of the most important staple crops in the world. China is one of the leading producers of potatoes, but the industry faces soilborne diseases such as Verticillium wilt. Most potato planting areas in China rotate the crop with sunflower which is also highly susceptible to Verticillium wilt. The comparison of the biological characteristics and pathogenicity of different mating types of *Verticillium dahliae* isolated from potato and sunflower in the major planting regions in China is of great importance. This is to help unravel the diversity in *V. dahliae* population and the sudden increase in infected fields. The diseased samples collected were cultured on PDA and the growing colony of pathogen isolated. Molecular techniques using specific primers were used to identify the *V. dahliae* pathogens and their mating type of the isolates obtained from the diseased sunflower and potato plants as well as their planting materials. The data obtained revealed that the dominant mating type population in sunflower was MAT1-1, whiles that of potato was MAT1-2, but Race 2 was the only race type identified for all the samples. There was a significant presence of MAT1-1 isolates present in potatoes, which is a new trend. Conventional crop rotation farming using sunflower is causing an increasing prevalence of MAT1-1 and mating type shift of isolates in potato in these regions.

## Introduction

A quarter of the world’s Potato (*Solanum tuberosum* L.) is produced from China, making it the largest producer of such an important crop globally^1^. Recently, potato has been listed as the fourth largest staple crop in China, after rice, wheat, and maize. Potato Verticillium Wilt caused by *Verticillium* spp. has progressively become a serious problem in the major potato-producing regions in China^2–5^. It may cause up to 50% yield losses^6^. *Verticillium dahliae,* one of the important species within the Verticillium genus, not only causes extensive yield losses in potato^7,8^ but also threatens many other dicotyledonous plants, such as cotton, lettuce, tomato, strawberry, pepper, and sunflower^9,10^.

Sunflower (*Helianthus annuus* L.) is one of the top oilseed crops grown for their edible oil. Sunflower seeds contain over 40% edible oil and 23% proteins and are good sources of fiber, vitamin E, copper, zinc, and B complex vitamins. Sunflower wilt caused by *V. dahliae* is a devastating disease threatening sunflower production worldwide^1^. *V. dahliae* is a soil-borne and seed-borne phytopathogenic fungus that causes wilt via the vascular system in many plant species^11^. It can form resting structures called microsclerotia that can survive in the soil for more than 20 years, thus making the control of the disease rather difficult. Methyl bromide fumigation was once used as an efficient way to control wilt caused by *V. dahliae,* but it has been banned due to its harmful environmental effect, which has made the control of Verticillium wilt on different hosts challenging^2^. Conventional control of *V. dahliae* through cultural practices such as crop rotation has proven limited due to the existence of cross-pathogenic isolates capable of infecting and surviving in several different plant species, including weeds^12,13^. There are some exceptions, however, such as the use of broccoli as a rotation crop, which has proven to be effective in reducing soilborne propagules and disease incidence in several cropping systems^14,15^.

*Verticillium dahliae* is a heterothallic fungus. In heterothallic fungi, sex compatibility is determined by a number of genes, one of which is the idiomorph of the MAT locus. The MAT1-1 idiomorph contains a gene that encodes an α-domain, while MAT1-2 contains a gene that encodes the high-mobility group (HMG) DNA-binding domain for mating proteins. In *V. dahliae,* only one idiomorph can be found in any one isolate, as it stands now^16,17^. The presence of both mating types could potentially lead to sexual reproduction, thus producing new sources of inoculum in the form of ascospores in the life cycle of *V. dahliae*. However, *V. dahliae* has been confirmed to reproduce only asexually on different hosts thus far.

In terms of race types, two different types, race 1 and race 2, have been confirmed among different isolates of *V. dahliae*^18^. Potato Verticillium wilt outbreaks on different Solanaceae cultivars on most farms have been caused by *V. dahliae* race 1^19^. During the 1950s, *Ve* genes were introduced into tomato, which provided farmers with new varieties that were resistant to race 1 strains^20^. In lettuce and cotton, both race 1 and race 2 have been identified; race 1 of *V. dahliae* was identified in potato isolates in Lebanon^21^, while potato isolates in China have been largely identified as race 2. In our laboratory, over the years, we identified all the *V. dahliae* strains isolated from sunflower as race 2 only. In some cases, two different race types of *V. dahliae* were identified on different hosts, except for sunflower^21–24^.

In this research, our aim was to find out whether the monotonous crop rotation between potato and sunflower was having any effect on the *V. dahliae* isolate population in these host crops across China and their pathogenicity characteristics. We isolated and identified *V. dahliae* from diseased potato and sunflower plants via Koch’s postulate and determined their race and mating types via molecular techniques using PCR with specific primers. The correlation between mating type, pathogenicity and virulence was also determined in this study.

## Materials and methods

### Sample collection

A total of 374 samples (exhibiting diseased basal stems) were collected from fields located in the Inner Mongolia Autonomous Region, Hebei Province, Shanxi Province, Shaanxi Province, Jilin Province, Liaoning Province and Heilongjiang Province (Supplemental Table 1). Among them, 167 samples were collected from potato and 207 from sunflower. The samples were stored in a 4 °C refrigerator before isolation. Apart from collecting diseased plants for pathogen isolation, some commercial sunflower seeds and potato tubers were also purchased from the market for *V. dahliae* isolation. All plant samples and seeds were collected in compliance with the regulations of the Chinese Academy of Sciences and all state laws regarding biological sample collection within the borders of China.

Disease samples collected from the various regions were done under the supervision and permission of the various extension officers of the Agriculture Academy of Sciences of Inner Mongolia Autonomous Region, Hebei Province, Shanxi Province, Shaanxi Province, Jilin Province, Liaoning Province and Heilongjiang Province and the individual farmers cultivating the land respectively for research purposes only.

### Culture media

Water agar medium (WA) used for the isolation of pathogenic fungi and single spore purification constituted 15 g of agar in 1000 mL of distilled water. For the cultivation of *V. dahliae* isolates, potato dextrose agar medium (PDA), which constituted 200 g potato, 20 g glucose, 15 g agar, and 1000 mL distilled water, was used, as well as complete medium (CM**),** which constituted 6 g yeast extract, 6 g caseinacid hydrolyzed, and 10 g sucrose in 1000 mL distilled water. These media were all freshly prepared and contained 100 mg/mL kanamycin (AMRESCO, Cat. No: K0408) to restrict the growth of contaminants.

### Isolation and purification of pathogens

The vascular tissues of the basal stem were cut vertically into 3–5 mm slices, dipped in 75% ethanol for 3–5 s, dipped in 0.1% NaClO for 30 s and rinsed twice with sterile double-distilled water. The slices were dried with sterilized filter paper in a lamina flow hood and then placed on water agar (WA) medium. Three days later, the edge of the growing colony surrounding the tissue slices was cut out and transferred onto a new PDA plate for culturing. The isolates cultured on PDA plates were washed with sterile water after the colony had grown to 2/3 of the petri dish. The conidiospore suspension was prepared with sterilized distilled water and adjusted to a concentration of 1.0 × 10^6^ conidia/mL using a hemocytometer. A 100 µL aliquot of the conidiospore suspension was drawn on freshly prepared WA medium, spread evenly and cultured at 25 °C for 2 days. The monospore colonies were cut and transferred onto PDA medium to obtain a pure culture.

The protocol above was also used for isolating pathogens from the sunflower seeds and tubers with a little modification. The seed hulls of sunflower seed samples were taken off, the radicles were destroyed (to prevent germination) then seeds were surface sterilized in 75% ethanol for 30 s then 0.1% NaClO for 30 s and rinsed twice with sterile double-distilled water for three minutes. The surface sterilized seeds were placed on sterilized filter paper and allowed to air dry before placing them on freshly prepared PDA media.

The potato tubers were cut across transversely into thin sheets of 2 mm, surface sterilized in 75% ethanol for 30 s then 0.1% NaClO for 1 min and rinsed twice with sterile double-distilled water for three minutes. The surfaced sterilized potato tuber samples were then placed on sterilized filter paper and allowed to air dry before they were cultured on freshly prepared PDA media.

### DNA isolation and PCR amplification

DNA extraction was carried out using the CTAB protocol as described by Doyle^25^. Isolates were cultured on PDA medium at 25 °C for 2 weeks and then scratched off the mycelium carefully from the medium into 2.0 mL Eppendorf tubes. The mycelium samples were placed in liquid nitrogen in preparation for tissue lysis using TissueLyser LT (QIAGEN Hilden, Germany). The powdered mycelium was mixed with extraction buffer (100 mM Tris–HCl pH 8.0; 20 mM EDTA-Na 2; 1.4 M NaCl; 2% cetyltrimethyl ammonium bromide and incubated at 65 °C for 30 min. Phenol–chloroform-isoamyl alcohol (25:24:1) was added and centrifuged. Isopropanol was used for DNA precipitation.

DNA was used as a template for PCR amplification with specific primers synthesized by Beijing Housheng Botai Technology Co., Ltd. and listed in Table 2. The 25 µl PCR system contained 1 µl of each primer (10 µM), 0.5 µl Taq DNA polymerase (Tiangen, Beijing, China), 2 µl of dNTPs (2.5 mM), 2.5 µL of 10X PCR buffer, 18 µl of distilled water and 2.0 µl of DNA template. PCR was performed in a Gene Pro Thermal Cycler (BIOER) with the following procedure for all primer pairs: 94 °C for 5 min, followed by 35 cycles of 94 °C for 40 s, 56 °C for 40 s, 72 °C for 40 s, and 72 °C for 10 min for extension.

The amplicons were separated on a 1.0% agarose gel stained with GelView (BioTeke, Beijing, China) and then observed and photographed under UV light. Amplicons were sent to Beijing Housheng Botai company for sequencing. Sequencing results were subjected to BLAST on the NCBI website and compared with the available data in GenBank to confirm the species of the isolates.

### Mating-type and physiological race identification

The purified isolates were cultured on PDA medium for 5–7 days at 25 °C. Their genomic DNA was extracted and amplified with specific primers (listed in Table 1) that could identify different mating types and physiological races of *V. dahliae,* which were isolated from both sunflower and potato.

**Table 1:** Primers used in this study.

Target amplicon	Primer name	Primer sequence (5’-3’)	Amplicon size(bp)
Defoliating type	D-F	CATGTTGCTCTGTTGACTGG	550
	D-R	GACACGGTATCTTTGCTGAA	550
Non-defoliating type	ND-F	ATCAGGGGATACTGGTACGAGA	1500
	ND-R	GAGTATTGCCGATAAGAACATG	1500
Race1 of *V. dahliae*	VdAve1-F	AAGGGGTCTTGCTAGGATGG	900
	VdAve1-R	TGAAACACTTGTCCTCTTGCT	900
Race 2 of *V. dahliae*	VdR2-F	ACTTAACGAAAGCATGCGC	260
	VdR2-R	CTTGACTTGCCGGCTCC	260
Mating type-1	MAT1-1-F	CGATCGATTCGGCAAGG	600
	MAT1-1-R	CAGTACATCCACCTGCTGGCC	600
Mating type-2	MAT1-2-F	CGGCCGCCATTCGCATCC	300
	MAT1-2-R	CATGCCTTCCATGCCATTAGGCC	300

Primer pairs: Defoliating type (D-F/D-R), Nondefoliating type (ND-F/ND-R), V. dahliae Ave1 gene (VdAve1-F/VdAve1-R), *V. dahliae* race 2 (VdR2-F/VdR2-R), Mating type-1 (MAT1-1-F/MAT1-1-R), Mating type-2 (MAT1-2-F/MAT1-2-R).

### Morphological comparison of two mating-type strains of *V. dahliae*

Four isolates from the different mating types, MAT1-1 (P48 and S11) and MAT1-2 (P50 and S12), of both potato (P48 and P50) and sunflower (S11 and S12) were randomly selected for macro- and micromorphological comparison. The selected isolates were cultured on freshly prepared PDA medium for two weeks. The cultured plates were washed with sterilized water to prepare conidiospore suspensions (1 × 10^6^ conidia/mL), and then 2 μL was pipetted onto the center of the PDA culture medium for smearing. The plates were kept in an incubator for 7 days at a temperature of 25 °C. The colony morphology was observed physically 7 days post inoculation (dpi). A conidiospore suspension was made to observe the conidia and hyphal structures using an optical microscope.

### Pathogenicity comparison among different isolates

Seedlings of both potato and sunflower were grown under greenhouse conditions and used to ascertain the pathogenicity of isolates of both MAT1-1 and MAT1-2 of *V. dahliae* recovered from both hosts. The selected isolates were cultured in CM media to produce the conidiospore suspension. We prepared 20 plants for each isolate for inoculation, and each plant was inoculated with 200 mL of conidiospore suspension (1 × 10^6^ conidia/mL) using the root dipping method^26^ (Alkher et al. 2009). 20 plants were inoculated with water as control. The entire experimental setup was repeated three times, and symptoms were recorded with the criteria listed in Table 2 after 21 dpi.

**Table 2:** Disease severity index of Potato and Sunflower Wilt.

Disease index scale	Potato			Sunflower	
	Value	Symptoms	Disease index scale	Value	Symptoms
1	0	Healthy plants	1	0	Healthy plants
2	1	up to 40% chlorosis and 1–20% necrosis	2	1	25% foliar chlorosis and stunting
3	2	up to 65% chlorosis and 21–35% necrosis	3	2	26–50% foliar chlorosis and stunting
4	3	100% chlorosis, 36–70% necrosis	4	3	51–75% foliar chlorosis and stunting
5	4	100% chlorosis, 71–100% necrosis	5	4	75%–100% severe leaf chlorosis and stunting along with plants death

The disease index was calculated according to the formula below^27^ (Xiao et al. 1998):

$$\begin{aligned} & {\text{Disease index}} = \\ & \quad \quad \frac{{100{*}\sum \left( {\text{number of diseased leaves in each scale rating * corresponding value in each rating}} \right)}}{{\left( {\text{total number of leaves examined * maximum rating value}} \right)}} \\ \end{aligned}$$

## Results

### Identification and mating type classification of *V. dahliae* isolated from both sunflower and potato

**Figure 1:**
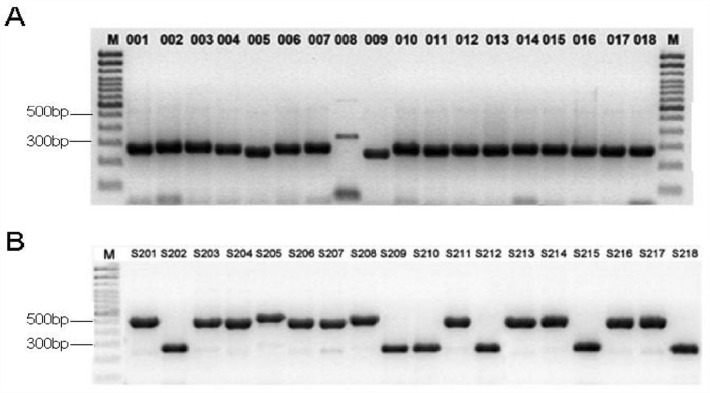
Molecular confirmation of sunflower and potato *V. dahliae* morphological mating types. PCR amplicons on gel after amplification with **a** MAT1-2F/R (upper) and MAT1-1F/R (below) specific primers for potato isolates and **b** MAT1-2F/R (upper) and MAT1-1F/R (below) specific primers for sunflower isolates. M; 100 bp DNA Ladder (110,488,058, Invitrogen, USA).

Samples including diseased plants, tubers (potato) and seeds (sunflower) were used for pathogen isolation. In total, 374 isolates were successfully obtained and confirmed as *V. dahliae* both morphologically and molecularly. Among them, 207 isolates were isolated from sunflower and 167 from potato samples. Mating types were classified through PCR using mating type-specific primers for *V. dahliae.* The amplicon results showed that both mating types, MAT1-1 and MAT1-2, were identified in the tested isolates recovered from both sunflower and potato (Fig. 1). MAT1-1 was the predominant mating type among isolates obtained from both the sunflower disease samples and seed coats, accounting for more than 70% of the total isolates recovered. Surprisingly, the isolates from the potato tubers and diseased samples were mainly dominated by the MAT1-2 mating type, making up 90%.

Among 89 isolates obtained from sunflower seeds, 68.54% were identified as MAT1-1, and 31.46% were MAT1-2, whereas the ratios of both MAT1-1 and MAT1-2 were 86.44% and 13.56%, respectively, among 118 tested isolates recovered from diseased sunflower plants. Regarding the 55 isolates recovered from potato tubers, all isolates were classified as MAT1-2; however, 112 isolates recovered from diseased potato plants, and the percentages of MAT1-1 and MAT1-2 were 16.96% and 83.04%, respectively, in contrast to the results obtained from the sunflower isolates (Table 3). Using a single factor ANOVA at (*p* ≤ 0.05) the p value calculated from the data collected in Table 3 was 0.9328 which proved there was no significant difference between the mating types of *V. dahliae* recorded in potato and sunflower samples.

**Table 3:** Identification of mating type ratio of Verticillium dahliae.

Pathogenic host	Number of strains of *V. dahliae*	Proportion of MAT1-1 (%)	Proportion of MAT1-2 (%)
Sunflower seeds	89	68.54	31.46
Sunflower plant	118	86.44	13.56
Potato tuber	55	0.00	100.00
Potato plant	112	16.96	83.04

**Figure 2:**
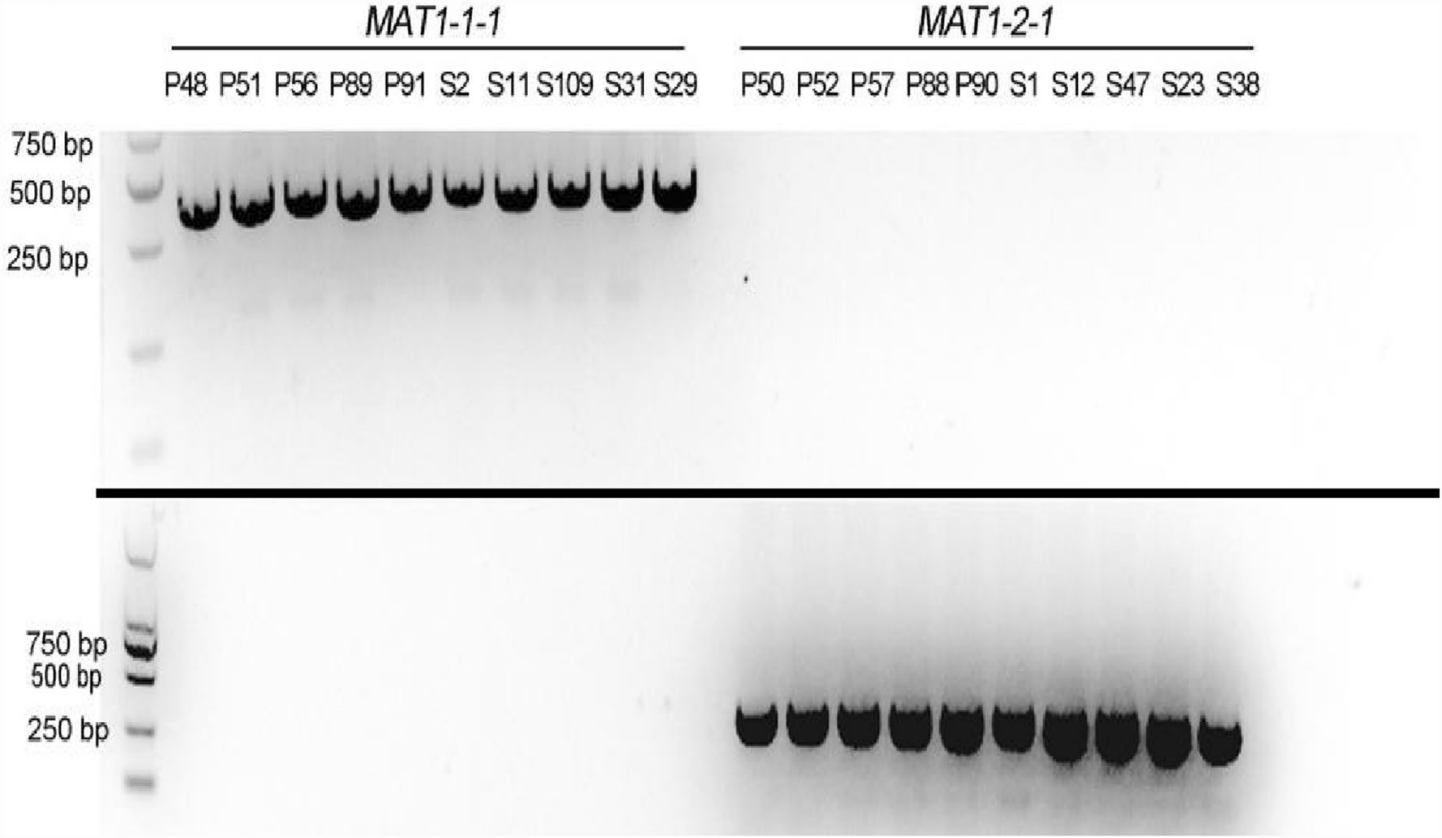
Reconfirmation of mating type of 20 randomly selected *V. dahliae* isolates using highly specific primers. M; Trans 2 K marker, left; shows all MAT1-1 isolates from both potato and sunflowers, right; shows all MAT1-2 isolates from both potato and sunflower.

Among all the tested isolates above, 20 of them were randomly selected, 10 from potato and 10 from sunflower, and subjected to molecular reconfirmation using specific primers of both mating types of *V. dahliae*. The results obtained buttressed that of the initial identification via PCR. MAT1-1 isolates from both hosts had an amplicon length of 600 bp, while that of MAT1-2 was 300 bp in both host samples (Fig. 2). Among the tested isolates, 10 MAT1-1 isolates, five each from potato and sunflower, and 10 MAT1-2 isolates five from both hosts were randomly selected and reconfirmed via PCR as MAT1-1 or MAT1-2 mating type separately (Fig. 2 and Table 3). Race type identification of *V. dahliae* isolated from both sunflower and potato were molecular identified as race 2 using race specific primers.

### Pathogenicity comparison on both mating types isolates

**Figure 3:**
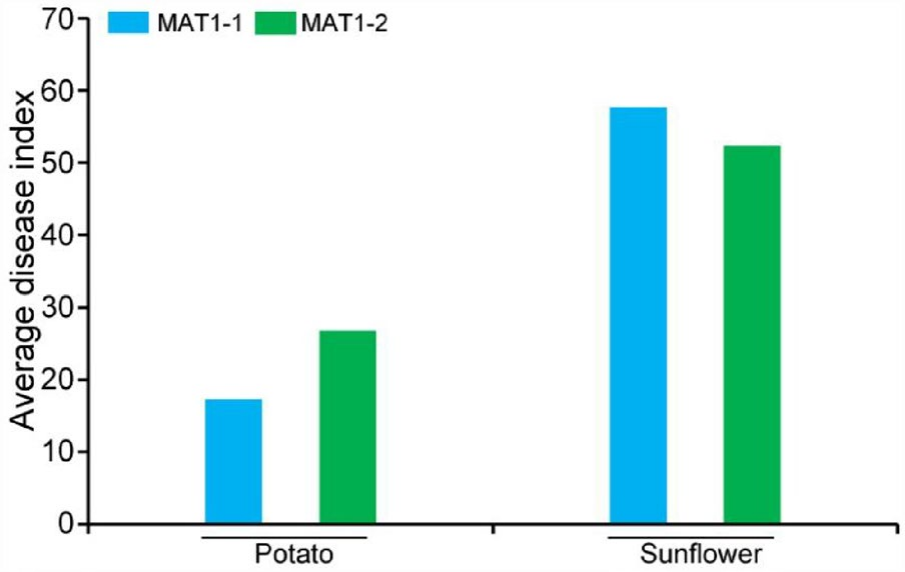
Average disease index of the 20 randomly selected *V. dahliae* isolates from potato and sunflower. MAT1-1 and MAT1-2 represent morphological mating types 1 and 2, respectively. The results were obtained 25 days post root dipping inoculation of host plants.

The 20 randomly selected isolates of different mating types, MAT1-1 and MAT1-2, previously mentioned were subjected for pathogenicity test on their respective host from which they were isolated initially. After 25 dpi, the disease index of the different mating type isolates obtained from the same host varied (Fig. 3). However, the average disease index of the MAT1-1 potato isolates was 17.29, while that of sunflower was 57.71. For the MAT1-2 isolates, the average disease index of both tested mating type isolates on their respective recovered hosts was recorded as 26.79 for potato and 52.59 for sunflower. This development shows the sunflower isolates were more virulent than the potato isolates, regardless of which mating type was being used for inoculation (Fig. 3).

### Comparative biological characterization of two different mating type isolate

**Figure 4:**
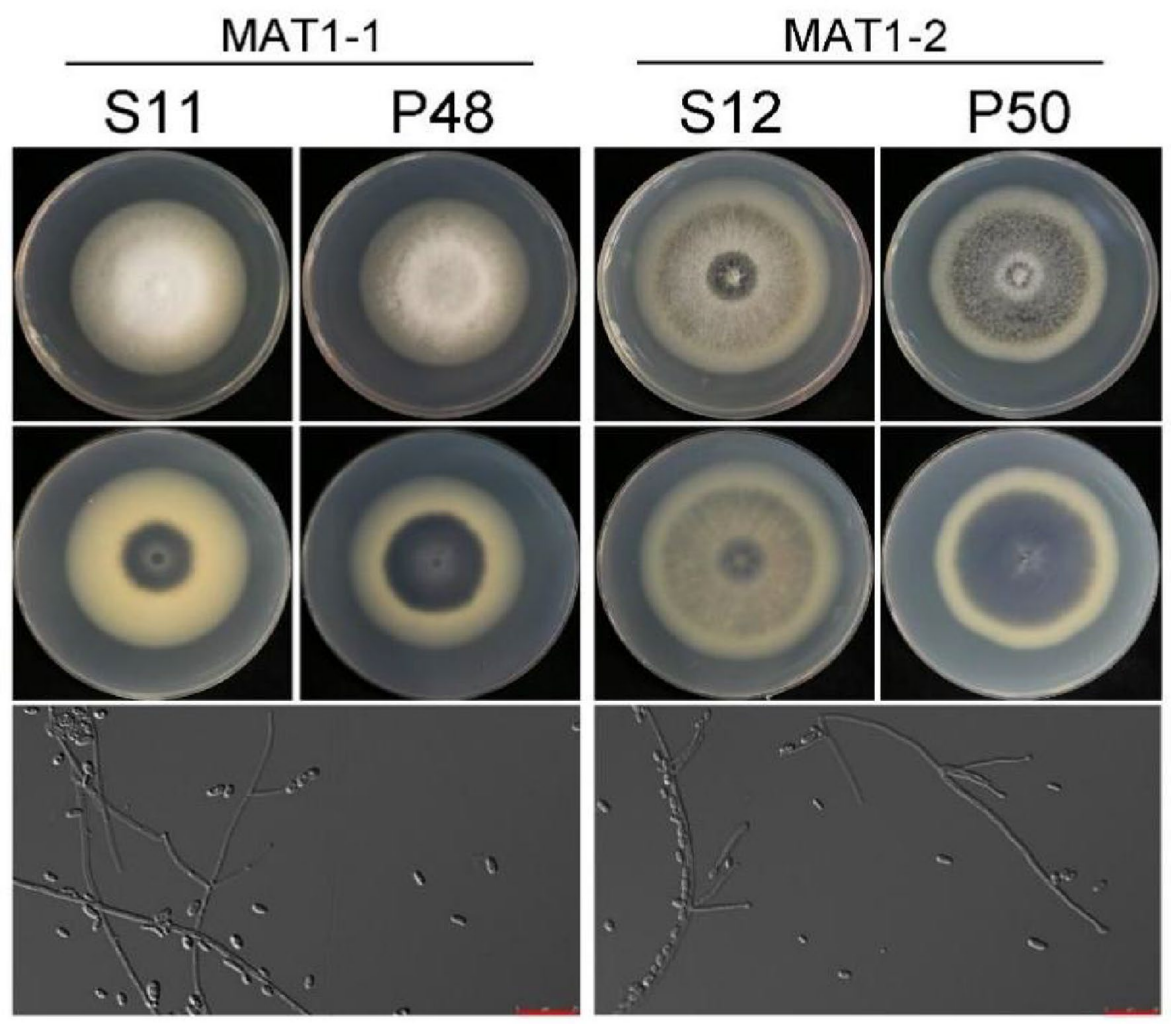
Physiological and morphological observation of *V. dahliae* isolates. Macro- and micro-observations of the two potato isolates. S11 and S12 were isolated from sunflower plants, while P48 and P50 were isolated from potato plants. Micro observation of isolates below has a magnification of 20 X.

Morphological comparison among four isolates, which were isolated from potato (P48 and P50) and sunflower (S11 and S12), and identified as different mating types MAT1-1 (P48 and S11) and MAT1-2 (P50 and S12). The different mating type isolates grew whitish hyphae after 7 days of culture. However, both the MAT1-1 strain, P48 from potato and S11 from sunflower, produced more whitish hyphae than the MAT1-2 strains P50 and S12. Potato strain P50 produced more melanin than strain P48; sunflower strain S11 produced a small amount of melanin around the inoculated plug, whereas more melanin was deposited in the medium by strain S12, which was also isolated from sunflower but identified as a different mating type. There were no significant differences in the average growth rate or the morphology of conidia and conidiophores (Fig. 4).

## Discussion

The samples collected in this experiment were inspired by the fact that most farmers in the northern part of China plant sunflower and potato in successive rotational farming. This has contributed to an increasing Verticillium wilt disease index in both sunflower and potato fields. Upon ascertaining the mating type of V. dahliae recovered from diseased samples through the use of mating type specific primers. PCR results showed both idiomorph mating types of *V. dahliae* present. The MAT1-2 idiomorph was dominant in the total tested isolates, which is in agreement with previous research carried out in other countries of the world concerning the* V. dahliae *mating type population^28^. The isolates recovered from potato had the highest number of MAT1-2 isolates compared to sunflower, which could be due to a number of factors. This is the first time a significant number of MAT1-2 isolates have been recovered from potato samples compared with previously carried out research on Potato Verticillium wilt in China^29^. To our surprise, no MAT1-1 isolates were recovered from the potato tubers, but 55 isolates of MAT1-2 were identified, indicating a certain level of resistance exists in potato tuber against *V. dahliae* MAT1-1 during the fruiting stage. Additionally, the limited sample size of isolates from potato tubers is another reason for this result. The impact of Verticillium wilt on potato was not much a concern until recently with the report of infected fields in the major potato production regions of China^2,28^. The continuous planting of crops in infected fields and the use of pathogen-contaminated planting materials have given rise to the increasing disease severity of *V. dahliae* in both sunflower and potato production regions. It is unclear why there were more MAT1-2 populations in the potato *V. dahliae* isolates, since conventional MAT1-1 populations are predominant in many crops, such as cotton and sunflower. The disease sample collection sites, where the mating type of most isolates was identified as MAT1-2, were located behind Yin mountain, where the temperature is mostly low throughout the year due to its topography. We hypothesize that the low average temperature in that region during summer may be the reason for the increasing *V. dahliae* MAT1-2 population in those potato fields, On the average, most of the sites behind Yin moutain had a temperature around 18–20 °C and an average precipitation of 36 mm during the cropping season; these environmental factors are much lower than that in region in front of Yin mountain. This might be due to the selection pressure for the development of *V.dahliae*. The environmental factors were conducive for microsclerotia deposit in the soil and plant materials, thus causing an increase in disease index in cropping season.

The two different mating types of *V. dahliae* found in those fields could be as a result of sexual interaction among the idiomorphs. Although the devastating effect of the MAT1-1 population has been extensively reported in sunflower^24^, the average disease index of MAT1-2 isolates recorded was almost equal to that of MAT1-1 (Fig. 3). Virulence among the different mating types of *V. dahliae* population tested by the disease index recovered within the sunflower population differed. The disease index of the two mating types of *V. dahliae* population in the sunflower was higher than that of the potato population; although the disease symptoms of MAT1-1 and MAT1-2 V*. dahliae* were similar. We observed high virulence in the sunflower isolates as compared to the potato isolates (Fig. 4). A significant number of MAT1-2 isolates recovered from Verticillium wilt-infected potatoes in China happened to be the first of its kind^24^. Several studies have reported an ever-increasing trend of race 2 relative to race 1, likely owing to its success in colonizing a greater variety of plants during crop rotation^18^. In this study, we also found that the MAT1-1 and MAT1-2 strains isolated from potato and sunflower were both race 2. This result is consistent with the previous identification results of the Sunflower Verticillium Wilt, but other research data have shown the presence of race1 in potato^21^.

## Conclusion

Verticillium wilt has recently been known to be an important disease in potato and sunflower cultivation in most farming regions in China. The results from this research will provide an in-depth understanding of the mating type(s) composition among *V. dahliae* causing potato and sunflower verticillium wilt. MAT1-1 has long been reported as the mating type isolate responsible for Verticillium wilt in most potato fields. A significant number of MAT1-2 isolates recovered from verticillium wilt-infected potato in China happens to be the first of its kind which raises lots of concern. There is an indication of an ever-increasing trend of race 2 relative to race 1 in host crops. The increasing prevalence of MAT1-1 and mating type shift of isolates in potato could be a result of conventional crop rotation farming being practiced in these regions, mostly using sunflower. The increasing spread of Verticillium wilt in China among different crops is worrying and must be managed and controlled effectively.

The results obtained in this study will alert researchers about the diversity occurring among *V. dahliae* populations. These data also provide important information for breeders and fungicide producing company to understand the dynamics in tackling Verticillium wilt in China and the world. We suggest new resistant cash crops be introduced by farmers into the crop rotation system in these regions to decrease *V. dahliae* inoculum in the soil.

## Supplementary Information

Supplementary Information.

## Data Availability

All data generated or analysed during this study are included in this published article (and its Supplementary Information files).
