# Supplementary material for: Identification and comparison of biological characteristics and pathogenicity of different mating types of V. dahliae isolated from potato and sunflower
Source: Sci Rep. 2022 Jul 27;12:12840. doi: 10.1038/s41598-022-17196-x (PMC9329468; doi:10.1038/s41598-022-17196-x)
Supplement: Supplementary file 1 — Supplementary Information. [file 41598_2022_17196_MOESM1_ESM.doc]

Supplemental Table 1. Information on isolates and mating type identification of *Verticillium dahliae* of sunflowers and potatoes

| **Isolate** | **Host** | **City or Location** | **Longitude** | **Latitude** | **Species** | **Mating Type** | **Year of Isolate** |
| --- | --- | --- | --- | --- | --- | --- | --- |
| P048 | Potato | Hunyuan-Datong | E113°41′56″ | N39°41′56″ | *Verticillium dahliae* | *MAT1-1-1* | 2017 |
| P050 | Potato | Hunyuan-Datong | E113°41′56″ | N39°41′56″ | *Verticillium dahliae* | *MAT1-2-1* | 2017 |
| P051 | Potato | Hunyuan-Datong | E113°41′56″ | N39°41′56″ | *Verticillium dahliae* | *MAT1-1-1* | 2017 |
| P052 | Potato | Hunyuan-Datong | E113°41′56″ | N39°41′56″ | *Verticillium dahliae* | *MAT1-2-1* | 2017 |
| P056 | Potato | Youyu-Shuozhou | E112°28′1″ | N39°59′21″ | *Verticillium dahliae* | *MAT1-1-1* | 2017 |
| P057 | Potato | Youyu-Shuozhou | E112°28′1″ | N39°59′21″ | *Verticillium dahliae* | *MAT1-2-1* | 2017 |
| P088 | Potato | Yijinhuoqi-Eerduosi | E109°19′56″ | N39°23′11″ | *Verticillium dahliae* | *MAT1-2-1* | 2016 |
| P089 | Potato | Yijinhuoqi-Eerduosi | E109°19′56″ | N39°23′11″ | *Verticillium dahliae* | *MAT1-1-1* | 2016 |
| P090 | Potato | Yijinhuoqi-Eerduosi | E109°19′56″ | N39°23′11″ | *Verticillium dahliae* | *MAT1-2-1* | 2016 |
| P091 | Potato | Yijinhuoqi-Eerduosi | E109°19′56″ | N39°23′11″ | *Verticillium dahliae* | *MAT1-1-1* | 2016 |
| S002 | Sunflower | Chayouhouqi-Wulanchabu | E113°11′58″ | N41°26′23″ | *Verticillium dahliae* | *MAT1-1-1* | 2015 |
| S001 | Sunflower | Chayouhouqi-Wulanchabu | E113°11′58″ | N41°26′23″ | *Verticillium dahliae* | *MAT1-2-1* | 2015 |
| S011 | Sunflower | Chayouhouqi-Wulanchabu | E113°11′58″ | N41°26′23″ | *Verticillium dahliae* | *MAT1-1-1* | 2015 |
| S012 | Sunflower | Chayouhouqi-Wulanchabu | E113°11′58″ | N41°26′23″ | *Verticillium dahliae* | *MAT1-2-1* | 2015 |
| S109 | Sunflower | Yulin | E109°44′5″ | N38°17′8″ | *Verticillium dahliae* | *MAT1-1-1* | 2011 |
| S047 | Sunflower | Yulin | E109°44′5″ | N38°17′8″ | *Verticillium dahliae* | *MAT1-2-1* | 2011 |
| S031 | Sunflower | Wulatezhongqi-Bayannaoer | E108°39′8″ | N40°44′12″ | *Verticillium dahliae* | *MAT1-1-1* | 2014 |
| S023 | Sunflower | Wulatezhongqi-Bayannaoer | E108°39′8″ | N40°44′12″ | *Verticillium dahliae* | *MAT1-2-1* | 2014 |
| S029 | Sunflower | Arongqi-Hulunbeier | E123°27′34″ | N48°07′33″ | *Verticillium dahliae* | *MAT1-1-1* | 2011 |
| S038 | Sunflower | Arongqi-Hulunbeier | E123°27′34″ | N48°07′33″ | *Verticillium dahliae* | *MAT1-2-1* | 2011 |
